# Warfarin anticoagulation in hemodialysis patients with atrial fibrillation: comparison of nephrologist-led and anticoagulation clinic-led management

**DOI:** 10.1186/s12882-017-0809-x

**Published:** 2018-01-08

**Authors:** Hamad Bahbahani, Ahmed AlTurki, Ahmed Dawas, Mark L. Lipman

**Affiliations:** 1 Division of Nephrology, Department of Medicine, McGill University Health Centre, Montreal, Canada; 2 Divisions of Internal Medicine and Cardiology, Department of Medicine, McGill University Health Centre, Montreal, Canada; 3 Faculty of Medicine, McGill University, Montreal, Canada; 4 Division of Nephrology, Department of Medicine, Jewish General Hospital, Montreal, Canada

**Keywords:** Hemodialysis, Atrial fibrillation, Warfarin, Time in therapeutic range, International normalized ratio (INR)

## Abstract

**Background:**

There is conflicting evidence of benefit versus harm for warfarin anticoagulation in hemodialysis patients with atrial fibrillation. This equipoise may be explained by suboptimal Time in Therapeutic Range (TTR), which correlates well with thromboembolic and bleeding complications. This study aimed to compare nephrologist-led management of warfarin therapy versus that led by specialized anticoagulation clinic.

**Methods:**

In a retrospective cohort of chronic hemodialysis patients from two institutions (Institution A: Nephrologist-led warfarin management, Institution B: Anticoagulation clinic-led warfarin management), we identified patients with atrial fibrillation who were receiving warfarin for thromboembolic prophylaxis. Mean TTRs, proportion of patients achieving TTR ≥ 60%, and frequency of INR testing were compared using a logistic regression model.

**Results:**

In Institution A, 16.7% of hemodialysis patients had atrial fibrillation, of whom 36.8% were on warfarin. In Institution B, 18% of hemodialysis patients had atrial fibrillation, and 55.5% were on warfarin. The mean TTR was 61.8% (SD 14.5) in Institution A, and 60.5% (SD 15.8) in Institution B (*p*-value 0.95). However, the proportion of patients achieving TTR ≥ 60% was 65% versus 43.3% (Adjusted OR 2.22, CI 0.65–7.63) and mean frequency of INR testing was every 6 days versus every 13.9 days in Institutions A and B respectively.

**Conclusions:**

There was no statistical difference in mean TTR between nephrologist-led management of warfarin and that of clinic-led management. However, the former achieved a trend toward a higher proportion of patients with optimal TTR. This improved therapeutic results was associated with more frequent INR monitoring.

## Background

Warfarin is a mainstay therapy for reducing stroke risk in atrial fibrillation [1–4]. The use of a target international normalized ratio (INR) between 2 and 3 decreases thromboembolic risks without a significant increase in the risk of major bleeding [1, 5, 6]. The risk of thromboembolic events is even higher in patients with advanced chronic kidney disease, when compared to the general population leading to a higher morbidity and mortality in this population [4, 7, 8]. There is conflicting evidence of benefit versus harm for anticoagulation in hemodialysis patients. Several studies have demonstrated decreased mortality and a decreased risk of stroke with warfarin [7–9]. In contrast, other studies reported an increased risk of bleeding, without a significant impact on ischemic stroke risk or mortality [10], and even an increased risk of stroke, whether ischemic or hemorrhagic [11, 12]. This equipoise is reflected in the guidelines published by various national and international societies, with some advocating a prominent role for warfarin in hemodialysis patients with atrial fibrillation [3], while others adopting a contrary position, in which they advise against the use of warfarin for primary thromboembolic prophylaxis in hemodialysis patients with atrial fibrillation [4, 13]. The Kidney Disease: Improving Global Outcomes, guidelines now state that routine anticoagulation of stage 5 chronic kidney disease patients with atrial fibrillation for primary prevention of stroke is not indicated [13].

Time in therapeutic range (TTR), as measured by the Rosendaal method [14], was found to correlate well with bleeding and thromboembolic complications, and a TTR of <60% has been associated with increased mortality, major bleeding and systemic embolism [15]. Hemodialysis patients on warfarin were found to have lower TTR compared to the general population, though data is limited. Two retrospective studies reported TTRs of 49.2% [16] and 45.1% [17]. In a prospective study, Genovesi et al. demonstrated an overall median TTR of only 54% in hemodialysis patients on warfarin [18]. This study also showed that an improved TTR was correlated with lower risk of hemorrhage. Therefore, it is possible that the advantages of warfarin are not demonstrated in hemodialysis patients, in part, due to the suboptimal TTRs.

Anticoagulation management directed by specialized anticoagulation clinics has been found to reduce thromboembolic and bleeding complications, as well as optimize TTR in atrial fibrillation patients [19]. Furthermore, a recent systematic review demonstrated superior anticoagulation management by pharmacists compared to usual medical care (i.e anticoagulation management directed by the primary care provider) [20]. However, data based on TTR does not exist for the hemodialysis population. This study compared nephrologist-led management of warfarin therapy in terms of achieved TTR and frequency of INR testing, to specialized thrombosis clinic-led management in chronic hemodialysis patients.

## Methods

### Study design and settings

This is a retrospective cohort study of chronic hemodialysis patients with atrial fibrillation receiving warfarin for the prevention of thromboembolic complications of atrial fibrillation in two university teaching hospitals; the McGill University Health Centre (MUHC) and the Jewish General Hospital (JGH), both located in Montreal, Canada. Between January 2015 and November 2016, patients receiving hemodialysis at the above institutions were screened for eligibility. At the MUHC, warfarin-based anticoagulation is managed by nephrologists, whereas at the JGH it is managed through an anticoagulation clinic led by hematologists. The study was approved by the Medical/Biomedical Research Ethics Committee of the West-Central Montreal Island Health Board.

### Study population and data collection

Patients were identified using the following inclusion criteria: a minimum age of 18 years, on maintenance hemodialysis for end-stage renal disease, a documented history of atrial fibrillation or atrial flutter, and on warfarin for a minimum of 3 months. Patients who were on hemodialysis during part of the study period but either died, received a renal transplant, or were transferred to another centre for hemodialysis were included in the study. Patients were included if their total duration of non-hospitalized INR testing while on warfarin was at least 140 days, to ensure sufficient INR data for TTR analysis.

INR values on warfarin were collected retrospectively for up to 1 year starting from the last encounter with the patient during the study period. INR values during hospital admissions lasting more than 2 days were excluded as were any INRs obtained within the first week post discharge. The following additional data were collected from dialysis charts and/or electronic medical records: demographics (age and gender), CHADS2 score, total duration on hemodialysis, relevant comorbidities (congestive heart failure, coronary artery disease, hypertension, type 2 diabetes mellitus, cerebrovascular accidents, valvular atrial fibrillation), medication use (antiplatelet agents, NSAIDs, total duration of out-patient antibiotic use), and total number of days of hospitalization during the study period.

### Study end points

The end points of the study were; 1) the mean TTR, based on the Rosendaal method, 2) the proportion of patients achieving TTR ≥ 60%, based on the Rosendaal method, 3) mean frequency of INR testing.

### Statistical analyses

The study cohort was stratified into two groups, Nephrologist-led management (Institution A) and Anticoagulation clinic-led management (Institution B). Continuous variables were expressed as mean ± standard deviation (SD) using paired t-test analysis, whereas, the Chi-square and Fisher exact tests were used to compare binary variables. TTR results from both institutions were fitted in a regression model using PROC GLM, and a logistic model using PROC LOGISTIC was used in the analysis of proportion of patients achieving TTR ≥ 60%. Adjustments for the following variables were performed when calculating *p*-values and Odds Ratio (OR): hemodialysis vintage, total duration of hospitalization, and total number of days of outpatient antibiotic use. All statistical analyses were performed using SAS version 9.4.

### Cost analysis

To evaluate cost effectiveness of the two strategies, we utilized the laboratory cost of INR assay (0.50 Canadian dollars per test) and the per test professional fee allocated for warfarin management to anti-coagulation clinic physician (12.50 Canadian dollars) applicable for the Province of Quebec, Canada. The nephrologist is not eligible for the professional fee.

## Results

**Fig. 1:**
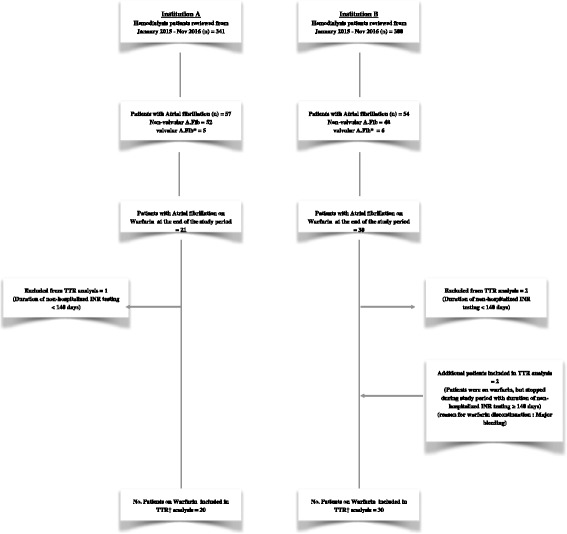
A diagram of the flow of the study. In Institution A: 57 out of 341 patients on chronic hemodialysis had a documented history of atrial fibrillation. Of whom, 52 patients had non-valvular Atrial fibrillation, and 5 had valvular atrial fibrillation. 21 patients were still on warfarin at their last encounter during the study period, in whom, 1 patient was excluded because duration of outpatient INR testing was <140 days. 20 patients were included in the final TTR analysis. In Institution B: 54 out of 300 patients on chronic hemodialysis had a documented history of atrial fibrillation. Of whom, 48 patients had non-valvular Atrial fibrillation, and 6 had valvular atrial fibrillation. 30 patients were still on warfarin at their last encounter during the study period, in whom, 2 patient were excluded because duration of outpatient INR testing was <140 days. 2 patients were added to the final TTR analysis, because they were on warfarin, but stopped during study period with duration of non-hospitalized INR testing ≥140 day. 30 patients were included in the final TTR analysis. *valvular atrial fibrillation: atrial fibrillation in patients with rheumatic mitral disease, mitral stenosis or prosthetic heart valves; †TTR: Time in Therapeutic Range

For the period starting from January 1st, 2015 and ending on November 26th, 2016, we identified 341 patients in Institution A and 300 patients in Institution B who were undergoing hemodialysis for at least 3 months. In Institution A, 57 patients (16.7%) had documented history of atrial fibrillation, of whom 21 (36.8%) were on warfarin at the end of the study period. In Institution B, 54 patients (18%) had history of atrial fibrillation, and 30 (55.5%) were on warfarin. Following the application of the inclusion criteria, the INR data of 20 patients in Institution A and 30 patients in Institution B were included in the final TTR analysis. A flow diagram of the study is illustrated in (Fig. 1).

### Patient characteristics

The baseline characteristics of patients included in the TTR analysis are shown in (Table 1). Mean age was 75.6 and 79.3 years in Institution A and Institution B, respectively, of which 60% were male in both institutions. The distribution of medical co-morbidities associated with increased stroke risk (such as previous CVA, type 2 DM, hypertension, congestive heart failure, and valvular atrial fibrillation), as well as CHADS2 scores were similar in both institutions. Hemodialysis patient vintage was also comparable (Institution A: mean 42.6 ± 39.6 months, Institution B: 50.8 ± 52.7 months). Despite the similar number of hospitalization events in patients from both institutions, the patients in Institution A had a shorter duration of hospitalization (mean 10.9 ± 15.6 days) compared to those in Institution B (mean 20 ± 36.4 days). However, this difference was not statistically significant. Moreover, the duration of outpatient antibiotic use was shorter in the patients of Institution A in comparison to those of Institution B (mean 12.5 ± 26.0 vs 27.1 ± 66.7 days, respectively), but the difference was not statistically significant.

**Table 1:** Baseline characteristics of hemodialysis patients on warfarin

	Institution A *n* = 20	Institution B *n* = 30	P-value
Age, yrs	75.6	79.3	0.25
Male, *n* (%)	12 (60)	18 (60)	1
Comorbidities, *n* (%):			
CHF	11 (55)	17 (56.7)	0.91
CAD	11 (55)	16 (53.3)	0.91
DM (Type 2)	9 (45)	17 (56.7)	0.42
CVA	8 (40)	8 (26.7)	0.32
HTN	16 (80)	27 (90)	0.42
Valvular A.Fib	5 (25)	6 (20)	0.74
HD Vintage, months (mean ± SD)	42.6 ± 39.6	50.8 ± 52.7	0.54
CHADS2 score (mean)	3.6	3.4	0.65
No. Hositalization events (mean)	0.8	1.13	0.27
Total duration of Hospitalization, days (mean ± SD)	10.9 ± 15.6	20 ± 36.4	0.23
Total duration of outpatient Antibiotic use, days (mean ± SD)	12.5 ± 26.0	27.1 ± 66.7	0.28

*CHF* congestive heart failure, *CAD* coronary artery disease, *HTN* hypertension, *DM* Diabetes Mellitus, *Valvular A.Fib* atrial fibrillation with rheumatic mitral disease, mitral stenosis or prosthetic heart valves, *HD* hemodialysis

### TTR analysis

The mean TTR, based on Rosendaal method, was 61.8% (SD 14.5) at Institution A, and 60.5% (SD 15.8) at Institution B (unadjusted *p*-value 0.76, adjusted *p*-value 0.95) (Table 2). The mean frequency of INR testing was every 6 days in Institution A versus every 13.9 days in Institution B.

**Table 2:** Mean TTR measures between Institution (A) and (B)

	Institution A (*n* = 20)	Institution B (*n* = 30)	Difference	Adjusted^a^ *P* - value
Rosendaal method, %	61.8	60.5	1.3	0.95

*TTR* Time in Therapeutic Range

^a^ Adjusted for HD vintage, total duration of hospital admission, and total duration of outpatient antibiotic use

**Fig. 2:**
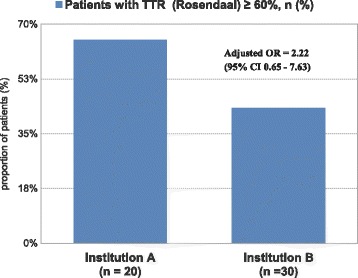
Graph showing proportion of patients achieving TTR (Time in Therapeutic range) ≥ 60%, based on Rosendaal method. Adjusted odds ratio (OR) of patients achieving TTR ≥ 60% in Institution A is 2.22 (95% CI 0.65–7.63)

Analysis of patients achieving the target TTR ≥ 60%, based on Rosendaal method, is illustrated in (Table 3) and (Fig. 2). At Institution A, 65% of hemodialysis patients taking warfarin achieved TTR ≥ 60%, whereas only 43.3% of patients at Institution B achieved that target level of TTR. The crude odds ratio of patients achieving TTR ≥ 60% at Institution A was 2.43 compared to Institution B. Even after adjustment for hemodialysis vintage, total duration of hospitalization, and total duration of outpatient antibiotic use, the odds ratio of patients achieving TTR ≥ 60% remained at 2.22 (95% CI 0.65–7.63).

**Table 3:** Odds ratio (OR) of patients with optimal TTR

	Institution A (*n* = 20)	Institution B (*n* = 30)	Crude Odds Ratio (OR)	Adjusted^a^ Odds Ratio (OR)	95% CI
Patients with TTR (Rosendaal) ≥ 60%, *n* (%)	13 (65)	13 (43.3)	2.43	2.22	0.65–7.63

^a^ Adjusted for HD vintage, total duration of hospital admission, and total duration of outpatient antibiotic use

### Cost analysis

The average annual number of INR tests was 60 in Institution A (nephrologist-led management) and 26 in Institution B (anti-coagulation clinic-led management). Therefore, at a total per test cost of 0.50$CAN, the average annual cost of INR testing per patient in Institution A is approximately 30.00 $CAD, whereas, at a total per test cost of 12.50$CAN, the average annual cost of INR testing per patient in Institution B is 338.00 $CAN. Therefore, there is significant overall cost savings associated with nephrologist-led warfarin management despite more frequent INR testing. It is important to note that this analysis is based on laboratory and professional fees specific to the Province of Quebec, Canada, which vary somewhat among Canadian provinces and more so across other countries.

## Discussion

Our study demonstrates a nearly equal mean TTR among chronic hemodialysis patients receiving warfarin, whether managed by specialized anticoagulation clinic or by nephrologists. In addition, both management modalities achieved mean TTRs that were superior to previously published data in hemodialysis patients, and comparable to those reported in general population. Interestingly, nephrologist-led warfarin management achieved more patients at the target TTR ≥60% than that achieved by anticoagulation clinic, with an odds ratio greater than 2. This outcome correlated with the higher frequency of INR testing that was observed with nephrologist-led management.

Time in Therapeutic Range (TTR), calculated by Rosendaal method, has been used as the optimal method to assess the effectiveness and safety of warfarin anticoagulation. It was found to correlate well with bleeding and thromboembolic complications. The data on TTR in the hemodialysis population is limited to a few studies that reported TTRs ranges between 49 and 54% [16, 18]. These results illustrate that TTRs in hemodialysis patients trend lower than other patient populations on warfarin for atrial fibrillation (55% - 64.5%) [21], and supports the contention that it is more challenging to optimize warfarin treatment in hemodialysis patients.

Hemodialysis patients with atrial fibrillation have a higher risk of thromboembolic events when compared to the general population with atrial fibrillation [7, 8]. The efficacy of warfarin in preventing thromboembolic events in hemodialysis patients with atrial fibrillation remains inconclusive [8–12, 22]. A large retrospective study by Shah et al., reported that warfarin use in dialysis patients was associated with 44% higher risk for bleeding without significant reduction of cerebrovascular events [10]. Moreover, Chan et al., described an increased risk of stroke with the use of warfarin in hemodialysis patients [11]. In contrast, a recent study by Brancaccio demonstrated a strong survival benefit with the use of warfarin in hemodialysis patients, with a 53% and 24% decreased mortality at 90 days and 6 years, respectively [9]. None of the above mentioned studies reported the TTR data in their study subjects. Currently, no clear explanation is forthcoming regarding the inconsistent impact of warfarin in hemodialysis patients with atrial fibrillation. This equipoise may be due, in part, to suboptimal TTRs typically achieved in this population. Therefore, it seems reasonable that efforts be directed towards improving TTR in hemodialysis patients to bring them into line with those achieved in other populations with atrial fibrillation, in whom warfarin treatment has been decidedly beneficial.

There is considerable evidence in the literature of general population with atrial fibrillation that achieved TTR can vary depending on the specialty of healthcare providers responsible for managing the warfarin therapy [19, 20]. However, the evidence of superiority for a specific provider-led management of anticoagulation in hemodialysis population is scarce. A study by Thomson et al. involving 64 outpatient hemodialysis patients compared nephrologist-led warfarin therapy to that of a nomogram-directed therapy managed by dialysis nurses, and showed that both strategies resulted in similar proportions of INR readings within target range, with less frequent INR testing associated with the nomogram-directed therapy [17]. However, this study did not analyze INR data using the Rosendaal method, which is the superior methodological standard to assess adequacy of warfarin anticoagulation [14, 15]. In our study, we observed that a higher proportion of patients had achieved therapeutic TTRs assessed by Rosendaal method with nephrologist-led management compared to anticoagulation clinic-led management, and that the nephrologist-directed management was associated with more frequent INR testing. The higher proportion of patients achieving target TTRs with nephrologist-led management may be due to the frequent and direct contact between nephrologists and hemodialysis patients. This dynamic facilitates timely and intimate knowledge of the patient’s clinical course (bleeding events, antibiotic exposure, adherence, etc.) which is often lacking in the case of the anticoagulation clinic-led management. The higher frequency of INR testing observed with the nephrologist-led management may be a consequence of this relationship or an independent contributor to improved results.

Our study has some noteworthy limitations. Firstly, the relatively small patient sample size and the inclusion of only two university-afflicted dialysis centers limits both the statistical power of the outcome measures and the broader inference to all hemodialysis patients. Secondly, the observational nature of this study may introduce the possibility of unmeasured bias. However, we did obtain data on many key factors that could influence TTR values in patients on warfarin, such as HD vintage, outpatient antibiotic use, and total duration of hospital admissions, and we adjusted for them in our TTR analysis.

Our study can be considered as a hypothesis- generating for a subsequent, larger, prospective study addressing whether improved TTRs can be widely achieved in hemodialysis patients, possibly through nephrologist-directed management of warfarin dosing, and whether improved therapy will translate into stroke reductions and less bleeding complications as seen in other patient populations with atrial fibrillation.

## Conclusion

There was no statistical difference of mean TTR between nephrologist-led management of warfarin to that of anticoagulation clinic-led management. However, the former achieved a trend of higher proportion of patients with optimal TTR which was associated with more frequent monitoring and greater cost effectiveness.

## Abbreviations

INR: International normalized ratio
JGH: Jewish General Hospital
MUHC: McGill University Health Centre
OR: Odds ratio
SD: Standard deviation
TTR: Time in therapeutic range
